# External validation of prognostic rules for early post-pulmonary embolism mortality: assessment of a claims-based and three clinical-based approaches

**DOI:** 10.1186/s12959-016-0081-5

**Published:** 2016-03-14

**Authors:** Erin R. Weeda, Christine G. Kohn, Gregory J. Fermann, W. Frank Peacock, Christopher Tanner, Daniel McGrath, Concetta Crivera, Jeff R. Schein, Craig I. Coleman

**Affiliations:** School of Pharmacy, University of Connecticut, 69 North Eagleville Road, Storrs, CT 06269 USA; University of Connecticut/Hartford Hospital Evidence-Based Practice Center, Hartford, CT USA; University of Saint Joseph School of Pharmacy, Hartford, CT USA; Department of Emergency Medicine, University of Cincinnati, Cincinnati, OH USA; Department of Emergency Medicine, Baylor College of Medicine, Houston, TX USA; Janssen Scientific Affairs LLC, Raritan, NJ USA

**Keywords:** Mortality, Pulmonary embolism, Prognosis, Risk assessment, Severity of illness index

## Abstract

**Background:**

Studies show the In-hospital Mortality for Pulmonary embolism using Claims daTa (IMPACT) rule can accurately identify pulmonary embolism (PE) patients at low-risk of early mortality in a retrospective setting using only claims for the index admission. We sought to externally validate IMPACT, Pulmonary Embolism Severity Index (PESI), simplified PESI (sPESI) and Hestia for predicting early mortality.

**Methods:**

We identified consecutive adults admitted for objectively-confirmed PE between 10/21/2010 and 5/12/2015. Patients undergoing thrombolysis/embolectomy within 48 h were excluded. All-cause in-hospital and 30 day mortality (using available Social Security Death Index data through January 2014) were assessed and prognostic accuracies of IMPACT, PESI, sPESI and Hestia were determined.

**Results:**

Twenty-one (2.6 %) of the 807 PE patients died before discharge. All rules classified 26.1–38.3 % of patients as low-risk for early mortality. Fatality among low-risk patients was 0 % (sPESI and Hestia), 0.4 % (IMPACT) and 0.6 % (PESI). IMPACT’s sensitivity was 95.2 % (95 % confidence interval [CI] = 74.1–99.8 %), and the sensitivities of clinical rules ranged from 91 (PESI)-100 % (sPESI and Hestia). Specificities of all rules ranged between 26.8 and 39.1 %. Of 573 consecutive patients in the 30 day mortality analysis, 33 (5.8 %) died. All rules classified 27.9–38.0 % of patients as low-risk, and fatality occurred in 0 (Hestia)-1.4 % (PESI) of low-risk patients. IMPACT’s sensitivity was 97.0 % (95%CI = 82.5–99.8 %), while sensitivities for clinical rules ranged from 91 (PESI)-100 % (Hestia). Specificities of rules ranged between 29.6 and 39.8 %.

**Conclusion:**

In this analysis, IMPACT identified low-risk PE patients with similar accuracy as clinical rules. While not intended for prospective clinical decision-making, IMPACT appears useful for identification of low-risk PE patient in retrospective claims-based studies.

**Electronic supplementary material:**

The online version of this article (doi:10.1186/s12959-016-0081-5) contains supplementary material, which is available to authorized users.

## Background

Guidelines suggest that patients with pulmonary embolism (PE) who are identified to have a low-risk of early post-PE all-cause mortality may be candidates for abbreviated hospital admission or outpatient treatment if appropriate follow-up can be arranged [1, 2]. Data from randomized trials and observational studies suggest that early discharge or outpatient treatment of low-risk PE patients is feasible and safe [3, 4].

A prior meta-analysis suggested at least one-third of acute PE patients could be classified as low-risk for early mortality according to clinical prediction rules [5]. This same meta-analysis identified the Pulmonary Embolism Severity Index (PESI), simplified PESI (sPESI) and Hestia clinical prediction rules as having high sensitivities and negative predictive values (NPVs) for identifying low-risk PE patients. In order to use PESI [6], sPESI [7] and Hestia [8] in the risk stratification of a patient with PE, access to vital signs, laboratory values, comorbid conditions and a cognitive evaluation at presentation is necessary. While PESI, sPESI and Hestia can be helpful in clinical practice, the extensive clinical data required to score these rules are not commonly found in claims databases or easily accessible to individual hospitals/health-systems. As a result, the utility of PESI, sPESI or Hestia for retrospective identification of low-risk patients with PE is limited.

While not originally derived to aid in prognostication in a prospective clinical setting, the In-hospital Mortality for Pulmonary embolism using Claims daTa (IMPACT) multivariable prediction rule utilizes data accessible within claims databases and/or claims from individual hospitals to retrospectively risk stratify patients with PE for early mortality [9]. Prior validation studies suggest IMPACT can accurately identify pulmonary embolism (PE) patients at low-risk of early mortality [10, 11]. The ability of IMPACT to correctly identify patients at low- and higher-risk of early mortality has not previously been compared to analogous clinical prediction rules. Therefore, using data from a single center, this study sought to externally validate IMPACT, PESI, sPESI and Hestia for predicting in-hospital and 30 day post-PE mortality.

## Methods

Preparation of this study report was in accordance with the Transparent reporting of a multivariable prediction model for individual prognosis or diagnosis (TRIPOD) statement [12]. For this retrospective cohort study, we identified consecutive patients diagnosed with acute PE between October 21, 2010 and May 12, 2015 using computerized claims records for admissions to Hartford Hospital (Hartford, Connecticut, USA). The hospital’s computerized claims system contains information on source of admission, International Classification of Diseases, Ninth Revision, Clinical Modification (ICD-9-CM) diagnosis and procedure codes, admission and discharge dates and discharge status. To be eligible for inclusion into this study, patients ≥18 years of age presenting to our institution had to have a primary diagnosis of PE (ICD-9-CM code = 415.1x). Consistent with prior studies, we excluded patients lacking objective confirmation of acute PE according to clinical guidelines. The following were considered confirmatory studies for the diagnosis of acute PE: high probability perfusion-ventilation lung scan (V/Q scan), computed tomography pulmonary angiography (CTPA) or pulmonary angiography diagnostic for PE, or a non-diagnostic V/Q scan or CTPA in combination with an abnormal compression ultrasonography of the lower extremities. Consistent with many prior studies of PE clinical prediction rules [5], subjects that received thrombolysis and/or pulmonary embolectomy within the 48 h of presentation were excluded as clinical guidelines do not consider such patients low-risk [1, 2]. All patients included in this study were managed according to usual clinical practice for our institution.

Risk stratification of patients with acute PE using IMPACT, PESI, sPESI and Hestia was performed according to published methods (Additional file 1) [6–9]. Patients with an IMPACT predicted mortality risk ≤1.5 % [9], PESI score ≤85 [6] or a sPESI or Hestia scores =0 [7, 8] were classified as low-risk for early mortality. Estimated mortality risk according to the claims-based IMPACT prediction rule [estimated % absolute risk = 1/(1 + exp(-x); where x = −5.833 + (0.026*age) + (0.402*myocardial infarction) + (0.368*chronic lung disease) + (0.464*stroke) + (0.638*prior major bleeding) + (0.298*atrial fibrillation) + (1.061*cognitive impairment) + (0.554*heart failure) + (0.364*renal failure) + (0.484*liver disease) + (0.523*coagulopathy) + (1.068*cancer)] was determined using all available hospital claims data (i.e., ICD-9-CM diagnosis and procedural codes) for each patient’s index PE encounter along with their age at time of presentation. ICD-9-CM coding for relevant IMPACT co-morbidities were performed according to the original IMPACT derivation paper [9]. Data necessary to classify patients as low- or high-risk of early mortality according to the PESI, sPESI and Hestia clinical prediction rules [6–8] were obtained by linking all included patients identified through hospital claims to the hospital’s electronic health record (EHR). We used vital signs (heart rate, blood pressure, respiratory rate, oxygen [O2] saturation, body temperature), laboratory values (serum creatinine, platelet count, total bilirubin) and mental status assessments obtained as close to the time of presentation for the index PE encounter as possible to score each of the clinical prediction rules [6]. For all patients admitted through the emergency department, the first vital sign, laboratory value and/or mental status assessment upon presentation (but within 24 h) was utilized. For patients directly admitted to the hospital we used the first values recorded on the day of hospital admission. Consistent with previous studies of this type, missing vital, laboratory and mental status assessment data were assumed to be normal [6]. For PESI, sPESI and Hestia, the presence of cancer, heart failure, chronic lung disease, severe liver disease (defined as a total bilirubin ≥2.5 mg/dL), heparin-induced thrombocytopenia and recent clinical events (gastrointestinal bleeding within 14 days, stroke with 4 weeks, surgery with 2 weeks) were assessed at time of hospital admission for the index PE encounter. All required data was abstracted from the electronic health record (including vital signs, laboratory values and emergency department, admission and consult notes) by trained study personnel blinded to study outcome.

All-cause in-hospital and 30 day post-PE mortality served as *a priori* endpoints for this study. In-hospital mortality was determined using the discharge status field for the index admission within the hospital billing system and electronic health record. Thirty-day mortality was based upon searches of the Social Security Death Index (SSDI) [11] performed >6 months after the last day of eligible inclusion in this analysis. Computerized health-system encounter data from our hospital’s billing records were queried for subsequent emergency visit claims, clinic visits and/or hospital readmissions outside of 30 days. These were used as confirmatory markers of vital status at 30 days. Beginning in March 2014, rules regarding access to data within the SSDI changed; restricting the release of three most recent years of data [13, 14]. For this reason, our 30 day mortality endpoint was assessed only in the subset of consecutive patients presenting to the hospital prior to January 31, 2014.

Baseline characteristics are described with means ± SDs for continuous data and counts and proportions for categorical data. Sensitivity, specificity and negative and positive predictive values for predicting early mortality were calculated for IMPACT, PESI, sPESI and Hestia along with 95 % confidence intervals (CIs). C-statistics were computed to evaluate each rule’s overall discriminative power. All database management and statistical analyses were performed using IBM SPSS Statistics version 22.0 (IBM Corp., Armonk, NY, USA). The study was approved by the Hartford Hospital institutional review board.

## Results

**Fig. 1:**
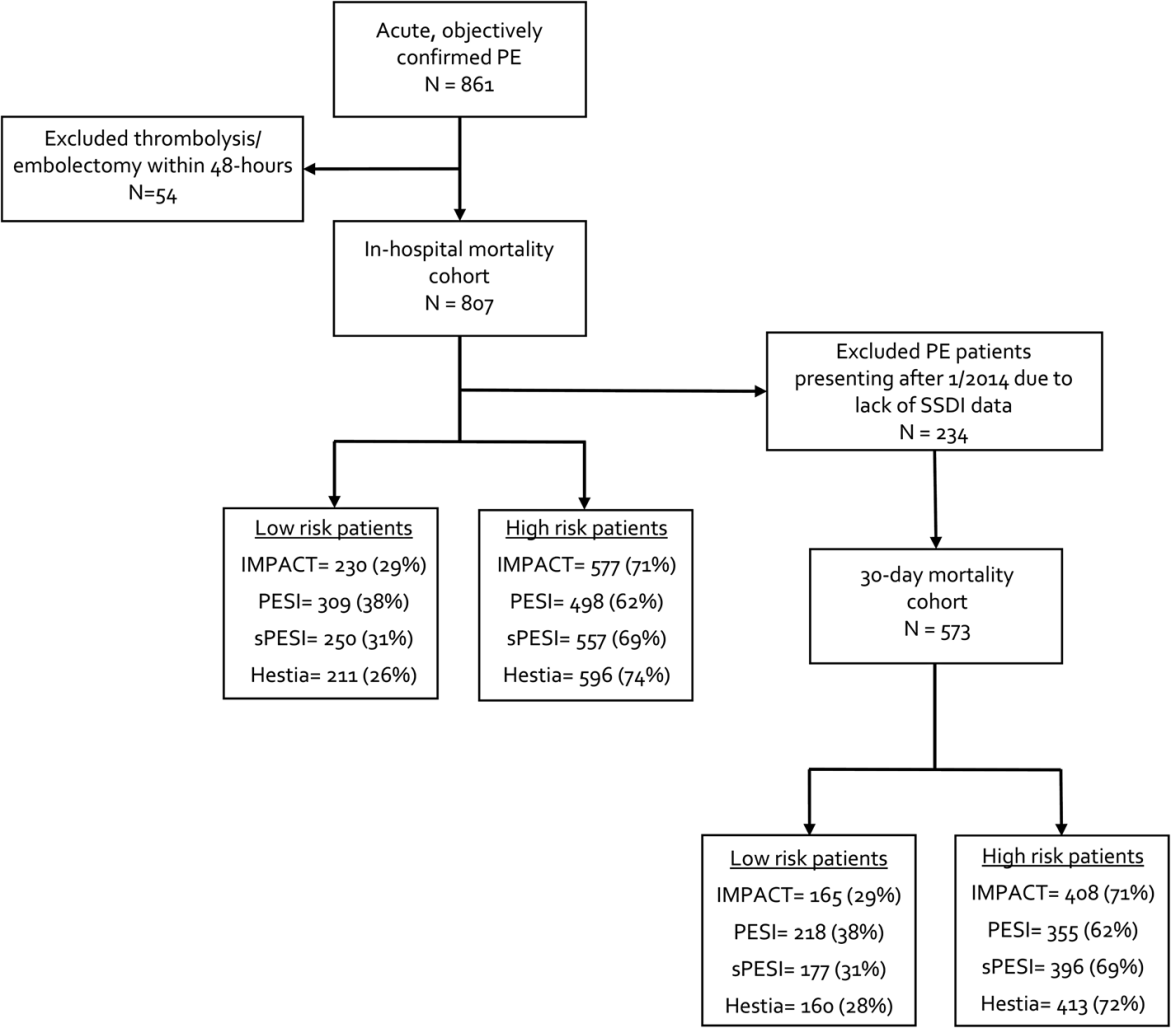
Identified PE patients and distribution of risk classes. IMPACT = hrs = hours; In-hospital Mortality for Pulmonary embolism using Claims daTa; PE = pulmonary embolism; PESI = Pulmonary Embolism Severity Index; SSDI = social security death index; sPESI = simplified Pulmonary Embolism Severity Index

A total of 861 patients with a primary ICD-9-CM code for acute PE and objective confirmation of diagnosis were identified (Fig. 1). Of these, 54 received thrombolytic therapy/embolectomy within 48 h of presentation, leaving 807 for analysis. Baseline characteristics of the cohort, stratified by low- and high-risk for each rule are shown in Table 1. Two-hundred and thirty-four patients presented with PE after January 2014 and were excluded from the 30 day mortality endpoint analysis, as SSDI data are not available for patients past this time point. The baseline characteristics of the 30 day mortality analysis patient subset were similar to the overall population (Additional file 1: Table S1).

**Table 1:** Characteristics of pulmonary embolism patients

Characteristic	Total Cohort^a^	IMPACT Low-Risk,	IMPACT Higher-Risk	PESI Low-Risk,	PESI Higher-Risk,	sPESI Low-Risk,	sPESI Higher-Risk	Hestia Low-Risk,	Hestia Higher-Risk,
	*N* (%)	*N* (%)	*N* (%)	*N* (%)	*N* (%)	*N* (%)	*N* (%)	*N* (%)	*N* (%)
	*N* = 807	*N* = 230	*N* = 577	*N* = 309	*N* = 498	*N* = 250	*N* = 557	*N* = 211	*N* = 596
Age (years, mean ± SD)	64.1 ± 16.47	46.4 ± 11.36	71.2 ± 12.38	51.9 ± 14.39	71.6 ± 12.76	56.3 ± 14.49	67.6 ± 16.12	59.9 ± 17.10	65.6 ± 16.00
> 80 years	145 (18.0)	0 (0)	145 (25.1)	9 (2.9)	136 (27.3)	0 (0)	145 (26.0)	27 (12.8)	118 (19.7)
Male gender	372 (46.1)	114 (49.6)	258 (44.7)	135 (43.7)	237 (47.6)	140 (56.0)	232 (41.7)	99 (46.9)	273 (45.8)
Cancer	254 (31.5)	18 (7.8)	236 (40.9)	15 (4.9)	239 (48.0)	0 (0)	254 (45.5)	51 (24.2)	203 (34.1)
Cancer (ICD-9-CM)	154 (19.1)	0 (0)	154 (26.7)	10 (3.2)	144 (28.9)	3 (1.2)	151 (27.1)	25 (11.8)	129 (21.6)
Chronic cardiopulmonary disease	230 (28.5)	29 (12.6)	201 (34.3)	42 (13.6)	188 (37.8)	0 (0)	230 (41.3)	37 (17.5)	193 (32.4)
Chronic lung disease	198 (24.5)	27 (11.7)	171 (29.6)	40 (12.9)	158 (31.7)	0 (0)	198 (35.5)	34 (16.1)	164 (27.5)
Chronic lung disease (ICD-9-CM)	224 (27.8)	22 (9.6)	202 (35.0)	47 (15.2)	177 (35.5)	7 (2.8)	217 (39.0)	36 (17.1)	188 (31.5)
Heart failure	62 (7.7)	2 (0.9)	60 (10.3)	3 (1.0)	59 (11.8)	0 (0)	62 (11.1)	4 (1.9)	58 (9.7)
Heart failure (ICD-9-CM)	75 (9.3)	1 (0.4)	74 (12.8)	9 (2.9)	66 (13.3)	5 (2.0)	70 (12.6)	8 (3.8)	67 (11.2)
Altered mental status at presentation	42 (5.2)	3 (1.3)	39 (6.8)	0 (0)	42 (8.4)	6 (2.4)	36 (6.5)	4 (1.9)	38 (6.4)
Cognitive impairment (ICD-9-CM)	60 (7.4)	0 (0)	60 (10.4)	7 (2.3)	53 (10.6)	6 (2.4)	54 (9.7)	7 (3.3)	53 (8.9)
Pulse (beats/min, mean ± SD)	93.2 ± 18.92	94.0 ± 18.51	92.9 ± 19.10	91.6 ± 17.35	94.1 ± 19.78	85.6 ± 13.38	96.6 ± 20.04	87.6 ± 17.73	95.2 ± 18.96
Pulse ≥ 110 beats/min	169 (20.9)	56 (24.3)	113 (19.6)	47 (15.2)	122 (24.5)	0 (0)	169 (30.3)	29 (13.7)	140 (23.5)
Systolic blood pressure (mmHg, mean ± SD)	133.6 ± 22.90	134.4 ± 21.75	133.3 ± 23.35	136.4 ± 21.32	131.9 ± 23.69	136.8 ± 21.60	132.2 ± 23.33	139.8 ± 22.30	131.4 ± 22.70
Systolic blood pressure <100 mmHg	36 (4.5)	7 (3.0)	29 (5.0)	3 (1.0)	33 (6.6)	0 (0)	36 (6.5)	3 (1.4)	33 (5.5)
O2 saturation (%, mean ± SD)	96.3 ± 3.50	96.8 ± 3.83	96.1 ± 3.34	96.3 ± 2.42	95.9 ± 3.97	97.0 ± 2.35	96.0 ± 3.87	97.1 ± 2.12	96.0 ± 3.83
O2 saturation <90 %	32 (4.0)	6 (2.6)	26 (4.5)	1 (0.3)	31 (6.2)	0 (0)	32 (5.7)	0 (0)	32 (5.4)
Oxygen needed to maintain O2 saturation >90 % for >24 h	412 (51.1)	79 (34.3)	333 (57.7)	112 (36.2)	300 (60.2)	90 (36.0)	322 (57.8)	0 (0)	412 (69.1)
Respiratory rate (breaths/min, mean ± SD)	19.1 ± 3.56	18.7 ± 3.31	19.3 ± 3.64	18.5 ± 2.20	19.5 ± 4.14	18.5 ± 2.51	19.4 ± 3.90	18.2 ± 2.37	19.5 ± 3.84
Respiratory rate ≥30 breaths/min	19 (2.4)	2 (0.8)	17 (2.9)	0 (0)	19 (3.8)	2 (0.8)	17 (3.1)	2 (0.9)	17 (2.9)
Temperature (degrees Celsius, mean ± SD)	97.6 ± 1.38	97.8 ± 1.29	97.6 ± 1.41	97.9 ± 1.38	97.4 ± 1.47	97.6 ± 1.28	97.6 ± 1.40	97.4 ± 1.24	97.7 ± 1.42
Temperature <36° Celsius	206 (25.5)	47 (20.4)	159 (27.6)	40 (12.9)	166 (33.3)	68 (27.2)	138 (24.8)	60 (28.4)	146 (24.5)
Thrombolysis or embolectomy > 48 h	4 (0.5)	2 (0.9)	2 (0.3)	3 (1.0)	1 (0.2)	3 (1.2)	1 (0.2)	0 (0)	4 (0.7)
High risk of bleeding^b^	101 (12.5)	37 (16.1)	64 (11.1)	48 (15.5)	53 (10.6)	37 (14.8)	64 (11.5)	0 (0)	101 (16.9)
PE on anticoagulation	62 (7.7)	17 (7.4)	45 (7.8)	21 (6.7)	41 (8.2)	11 (4.4)	51 (9.2)	0 (0)	62 (10.4)
History of heparin-induced thrombocytopenia	5 (0.6)	1 (0.4)	4 (0.7)	2 (0.6)	3 (0.6)	2 (0.8)	3 (0.5)	0 (0)	5 (0.8)
Medical or social reason for admission^c^	237 (29.3)	43 (18.7)	194 (33.6)	64 (20.7)	173 (34.7)	42 (16.9)	195 (34.9)	0 (0)	237 (39.8)
Need for intravenous pain medication for > 24 h	87 (10.8)	43 (18.7)	44 (7.6)	48 (15.5)	39 (7.8)	32 (12.8)	55 (9.9)	0 (0)	87 (14.6)
Severe liver impairment^d^	10 (1.2)	3 (1.3)	7 (1.2)	3 (1.0)	7 (1.4)	3 (1.2)	7 (1.3)	0 (0)	10 (1.7)
Liver disease (ICD-9-CM)	6 (0.7)	0 (0)	6 (1.0)	3 (1.0)	3 (0.6)	2 (0.8)	4 (0.7)	0 (0)	6 (1.0)
Creatinine clearance <30 mL/min	31 (3.8)	2 (0.9)	29 (5.0)	11 (3.6)	20 (4.0)	7 (2.8)	24 (4.3)	0 (0)	31 (5.2)
Renal failure (ICD-9-CM)	60 (7.4)	2 (0.9)	58 (10.1)	18 (5.8)	42 (8.4)	16 (6.4)	44 (7.9)	8 (3.8)	52 (8.7)
Hemodynamically unstable^e^	94 (11.6)	24 (10.4)	70 (12.1)	30 (9.7)	64 (12.9)	17 (6.8)	77 (13.8)	0 (0)	94 (15.8)
Myocardial infarction (ICD-9-CM)	40 (5.0)	4 (1.7)	36 (6.2)	15 (4.9)	25 (5.0)	16 (6.4)	24 (4.3)	2 (0.9)	38 (6.4)
Cerebrovascular disease (ICD-9-CM)	11 (1.4)	0 (0)	11 (1.9)	5 (1.6)	6 (1.2)	4 (1.6)	7 (1.3)	2 (0.9)	9 (1.5)
Prior major bleeding (ICD-9-CM)	28 (3.5)	1 (0.4)	27 (4.7)	8 (2.6)	20 (4.0)	5 (2.0)	23 (4.1)	3 (1.4)	25 (4.2)
Atrial fibrillation (ICD-9-CM)	87 (10.8)	2 (0.9)	85 (14.7)	15 (4.9)	72 (14.5)	11 (4.4)	76 (13.6)	18 (8.5)	69 (11.6)
Coagulopathy (ICD-9-CM)	41 (5.1)	3 (1.3)	38 (6.6)	11 (3.6)	30 (6.0)	12 (4.8)	29 (5.2)	6 (2.8)	35 (5.9)

^a^Of the 807 patients, 3 (0.4 %) patients had unknown values for respiratory rate; 2 (0.2 %) for pulse, systolic blood pressure, O2 saturation, and temperature; 516 (63.9 %) for bilirubin (component of liver disease); 1 (0.1 %) for platelets (component of bleed risk); and 1 (0.1 %) for glomerular filtration rate (creatinine clearance estimate)

*Hrs* hours, *ICD-9-CM* International Classification of Diseases-Ninth Revision-Clinical Modification, *min* minutes, *SD* standard deviation, *O2* oxygen

^b^Gastrointestinal bleeding in the preceding 14 days, stroke in the preceding 4 weeks, procedure in the preceding 2 weeks, bleeding disorder or thrombocytopenia (platelet count < 75 × 109/L), or uncontrolled hypertension (systolic blood pressure > 180 mmHg or diastolic blood pressure > 110 mmHg)

^c^Medical or social reason for hospital treatment was determined by trained study personnel using all data available in the electronic health record including vital signs, laboratory values, and emergency department, admission and consult notes

^d^Cirrhosis or bilirubin > 2.5 mg/dL

^e^Pulse ≥ 100 beats/minute and systolic blood pressure ≤ 100 mmHg or condition requiring admission to an intensive care unit

The IMPACT, PESI, sPESI and Hestia scores (mean ± SD) for the complete cohort of 807 PE patients were 3.9 ± 4.3, 96.4 ± 33.3, 1.1 ± 0.9 and 1.3 ± 1.1, respectively. While mean age for patients was 64.1 ± 16.57; when dichotomized into risk groups, high-risk patients were considerably older, regardless of prediction rule used. At time of presentation, mean vital sign values were within normal ranges in the overall analysis population; but more than half of the subjects required O2 supplementation to maintain saturations >90 %. The use of thrombolysis and/or embolectomy after 48 h was infrequent, occurring in only 4 (0.5 %) of patients.

The overall incidence of all-cause in-hospital mortality was 2.6 % (21/807). IMPACT, PESI, sPESI and Hestia classified 26 % (Hestia)-38 % (PESI) of the cohort as low-risk for early post-PE mortality. Fatality among low-risk patients was low (0–0.6 %), corresponding to NPVs of 99.4 (PESI)-100 % (sPESI and Hestia) (Table 2). IMPACT’s sensitivity was 95.2 % (95 % CI = 74.1–99.8 %), and the sensitivities of clinical rules ranged from 91 (PESI)-100 % (sPESI and Hestia). Specificities of all rules ranged between 26.8 (Hestia)-39.1 % (PESI) and C-statistics from 0.76 (sPESI)-0.86 (Hestia). Additional file 1: Table S2 describes the characteristics of patients who died in-hospital and had discordant risk categorization across any of the four prediction rules.

**Table 2:** Prognostic test characteristics for in-hospital mortality

	IMPACT	PESI	sPESI	Hestia
Low-Risk Mortality	1/230	2/309	0/250	0/211
n/N (%)	(0.4 %)	(0.6 %)	(0 %)	(0 %)
High-Risk Mortality	20/577	19/498	21/557	21/596
n/N (%)	(3.5 %)	(3.8 %)	(3.8 %)	(3.5 %)
Sensitivity	95.2 %	90.5 %	100 %	100 %
(95 % CI)	(74.1–99.8 %)	(68.2–98.3 %)	(80.8–100 %)	(80.8–100 %)
Specificity	29.1 %	39.1 %	31.8 %	26.8 %
(95 % CI)	(26.0–32.5 %)	(35.6–42.6 %)	(28.6–35.2 %)	(23.8–30.1 %)
PPV	3.5 %	3.8 %	3.8 %	3.5 %
(95 % CI)	(2.2–5.4 %)	(2.4–6.0 %)	(2.4–5.8 %)	(2.2–5.4 %)
NPV	99.6 %	99.4 %	100 %	100 %
(95 % CI)	(97.2–100 %)	(97.4–99.9 %)	(98.1–100 %)	(97.8–100 %)
C-statistic	0.766	0.792	0.762	0.857
(95 % CI)	(0.685–0.848)	(0.696–0.889)	(0.682–0.842)	(0.796–0.918)

*CI* confidence interval, *IMPACT* In-hospital Mortality for Pulmonary embolism using Claims data, *NPV* negative predictive value, *PESI* Pulmonary Embolism Severity Index, *PPV* positive predictive value, *sPESI* simplified Pulmonary Embolism Severity Index

Among the subset of 573 patients accessible within the SSDI, 33 (5.8 %) died of any cause within 30 days of presentation for PE. All rules classified 27.9 (Hestia)-38.0 % (PESI) of PE patients as low-risk, and fatality occurred in 0.0 % (Hestia)-1.4 % (PESI) of low-risk patients (NPVs = 98.6–100 %) (Table 3). IMPACT’s sensitivity for predicting 30 day mortality was 97.0 % (95%CI = 82.5–99.8 %), while sensitivities for clinical rules ranged from 91 (PESI)-100 % (Hestia). Specificities of rules ranged between 29.6 % (Hestia)-39.8 % (PESI) and their C statistics ranged from 0.73 (sPESI)-0.81 (PESI). Additional file 1: Table S3 describes the characteristics of patients who died within 30 days of presentation and had discordant risk categorization across any of the 4 prediction rules.

**Table 3:** Prognostic test characteristics for 30 day mortality

	IMPACT	PESI	sPESI	Hestia
Low-Risk Mortality	1/165	3/218	1/177	0/160
n/N (%)	(0.6 %)	(1.4 %)	(0.6 %)	(0 %)
High-Risk Mortality	32/408	30/355	32/396	33/413
n/N (%)	(7.8 %)	(8.5 %)	(8.1 %)	(8.0 %)
Sensitivity	97.0 %	90.9 %	97.0 %	100 %
(95 % CI)	(82.5–99.8 %)	(74.5–97.6 %)	(82.5–99.8 %)	(87.0–100 %)
Specificity	30.4 %	39.8 %	32.6 %	29.6 %
(95%CI)	(26.6–34.5 %)	(35.7–44.1 %)	(28.7–36.8 %)	(25.8–33.7 %)
PPV	7.8 %	8.5 %	8.1 %	8.0 %
(95 % CI)	(5.5–11.0 %)	(5.9–12.0 %)	(5.7–11.3 %)	(5.6–11.1 %)
NPV	99.4 %	98.6 %	99.4 %	100 %
(95 % CI)	(96.2–100 %)	(95.7–99.6 %)	(96.4–100 %)	(97.1–100 %)
C-statistic	0.804	0.805	0.731	0.791
(95 % CI)	(0.749–0.859)	(0.731–0.879)	(0.653–0.810)	(0.721–0.860)

*CI* confidence interval, *IMPACT* In-hospital Mortality for Pulmonary embolism using Claims data, *NPV* negative predictive value, *PESI* Pulmonary Embolism Severity Index, *PPV* positive predictive value, *sPESI* simplified Pulmonary Embolism Severity Index

## Discussion

In this analysis, the claims-based IMPACT prediction rule displayed prognostic accuracy similar to that of commonly used clinical risk stratification rules, including PESI and sPESI (which have been prospectively validated for identification of low-risk PE patients) and the Hestia criteria (which was prospectively designed to identify patients whom could be treated as outpatients). The 4 rules evaluated in this study classified between 1/4th and 2/5ths of patients as low-risk. Each exhibited sensitivities >90 %, NPVs >98.6 % and specificities <40 % for predicting in-hospital or 30 day all-cause mortality, and these findings are consistent with prior derivation and validation studies. To our knowledge, this is the first external validation study of Hestia [5, 9–11]. Taken together, our results suggests each of the four rules can identify a cohort of low-risk PE patients whom are unlikely to die within the first 30 days of presentation. However, because a minority of patients with PE (<6 % in our study) die within 30 days, these prognostic rules classify a substantial number of patients who ultimately survive into higher-risk groups (hence their lower specificities). Prognostic tests seldom have both high sensitivity and specificity. Therefore, when using a prognostic test to decide upon implementing a less conservative treatment strategy (e.g., discharging a patient with acute PE directly from the emergency department) higher sensitivity and NPV values are preferable.

The American College of Chest Physicians and the European Society of Cardiology guidelines support early discharge and/or home treatment of PE patients at low-risk for early mortality as long as they have adequate home circumstances [1, 2]. These guidelines suggest that clinicians use validated clinical prediction rules to assist in identification and selection of low-risk patients. Although in this analysis IMPACT displayed a similar ability to identify low-risk PE patients as PESI, sPESI and Hestia; IMPACT was not originally derived or validated to assist in prospective clinical decision-making (and is relatively more computationally complex compared to clinical prediction rules), and it should not be used for individual patient decision-making [9]. However, IMPACT appears valid for retrospective identification of low-risk PE patients and therefore could be used to aid in the performance of real-world outcomes studies and to enable payer/institution benchmarking of rates of low-risk PE patients treated at home or following an abbreviated admission. Using claim s data as described by IMPACT may have advantages over obtaining highly granular clinical information from the EHR (including reduced time and effort requirements).

Our study has limitations that require consideration. First, this validation study was performed retrospectively and therefore may be subject to biases, particularly due to missing data. Nonetheless, our study had similar rates of missing data than reported in prior prospective derivation/external validation papers of clinical prediction rules [5–7]. Second, this was a single-center study limiting its generalizability and sample size. However, baseline characteristics and mortality rates were consistent with national estimates [10, 15] and our sample size (573–807 patients) was large relative to many previously published external validation studies of PE clinical prediction rules [5]. Third, we could not assess 30 day mortality in our entire study cohort due to restrictions on the availability of SSDI data [14]. Despite this, the sample size of patients with objectively confirmed PE available for final analysis was robust. Moreover, we are unaware of any programmatic changes in evaluation and treatment of patients with PE at our institution since February 2014. Consequently, the likelihood of selection bias resulting from the unavoidable exclusion of patients after this date is low. Next, the more subjective nature of certain criteria in Hestia (i.e., medical or social reason for hospital admission and the need for intravenous pain medication for >24 h), make retrospective scoring challenging. This being said, the proportion of patients in our study classified as higher-risk because of these “subjective” criteria was not inconsistent with the Hestia derivation study [8]. Lastly, the 48 h cut-off used to exclude patients undergoing thrombolysis and/or embolectomy is somewhat arbitrary. We excluded patients receiving thrombolysis and/or embolectomy in less than 48 h because such patients likely had hemodynamic instability at presentation and would not be considered low-risk per guidelines [1, 2]. Of note, numerous studies evaluating the prognostic accuracy of clinical prediction rules have similarly excluded patients undergoing early thrombolysis and/or embolectomy [5]. However, when these procedures are performed later in a hospital stay (day 3 onwards), they are more likely an indicator of a therapeutic failure resulting in a poor clinical course (i.e., respiratory failure or cardiac arrest). In addition, the need for and timing of thrombolysis and/or embolectomy can easily be detected in a claims database and a clinical setting, allowing it to be implemented in our evaluation of IMPACT and clinical prediction rules. The 48 h cut-off was chosen *a priori* to approximate the likely timing used for assessing the similar Hestia criterion (i.e., the Hestia study required discharge within 24 h of PE diagnosis, likely resulting in the assessment of the 11 Hestia criteria within 48 h of initial PE presentation) [8].

## Conclusion

IMPACT identified low-risk PE patients with similar accuracy as PESI, sPESI and Hestia. While not designed for prospective clinical decision-making, IMPACT appears useful for identification of low-risk PE patient in retrospective claims-based studies.

### Ethics approval and consent to participate

This study was approved by the Hartford Hospital institutional review board with a waiver of informed consent.

### Consent for publication

Consent for publication: not applicable.

## Additional file

Additional file 1: Description of the IMPACT, PESI, sPESI and Hestia Prediction Rules. **Table S1.** Characteristics of Pulmonary Embolism Patients in the 30-Day Mortality Cohort. **Table S2.** Description of Patients Whom Died In-Hospital and Had Discordant Risk Categorization. **Table S3.** Description of Patients Whom Died After Discharge and Within 30-days of Presentation and Had Discordant Risk Categorization. (DOCX 35 kb)

## Abbreviation

CI: Confidence intervals
CTPA: Computed tomography pulmonary angiography
EHR: Electronic health record
ICD-9-CM: International classification of diseases, ninth revision, clinical modification
IMPACT: In-hospital mortality for pulmonary embolism using claims daTa
NPV: Negative predictive value
O2: Oxygen
PE: Pulmonary embolism
PESI: Pulmonary embolism severity index
PPV: Positive predictive value
sPESI: Simplified pulmonary embolism severity index
SSDI: Social security death index
TRIPOD: Transparent reporting of a multivariable prediction model for individual prognosis or diagnosis
USA: United States of America
V/Q: Ventilation/perfusion
