# Supplementary material for: External validation of prognostic rules for early post-pulmonary embolism mortality: assessment of a claims-based and three clinical-based approaches
Source: Thromb J. 2016 Mar 14;14:7. doi: 10.1186/s12959-016-0081-5 (PMC4790043; doi:10.1186/s12959-016-0081-5)
Supplement: Additional file 1: — Description of the IMPACT, PESI, sPESI and Hestia Prediction Rules. Table S1. Characteristics of Pulmonary Embolism Patients in the 30-Day Mortality Cohort. Table S2. Description of Patients Whom Died In-Hospital and Had Discordant Risk Categorization. Table S3. Description of Patients Whom Died After Discharge and Within 30-days of Presentation and Had Discordant Risk Categorization. (DOCX 35 kb) [file 12959_2016_81_MOESM1_ESM.docx]

**SUPPLEMENTAL MATERIAL**

**Additional file 1 Description of the IMPACT, PESI, sPESI and Hestia Prediction Rules**

| **These variables were used by IMPACT to estimate a logistic regression model to predict early mortality**^‡^**:**  *f(x) = 1/(1+exp(-x); where x = -5.833+(0.026*age)+(0.402*myocardial infarction)+(0.368*chronic lung disease) +(0.464*stroke)+(0.638*prior major bleeding)+(0.298*atrial fibrillation)+(1.061*cognitive impairment)+(0.554*heart failure)+(0.364*renal failure)+(0.484*liver disease)+(0.523*coagulopathy)+ (1.068*cancer)*  ^‡^Low risk is an estimated in-hospital mortality of <1.5%. |
| --- |

**In-hospital Mortality for Pulmonary embolism using Claims daTa (IMPACT) prediction rule**

The 11 comorbidities in the above equation were calculated based only upon the maximum of 25 ICD-9-CM diagnosis codes and procedural codes reported for each discharge. When possible, the presence or absence of comorbidities were determined using AHRQ’s 29-comorbidity index.

| **Variable** | **PESI Score*** |
| --- | --- |
| Age | Age in years |
| Male sex | +10 |
| History of cancer | +30 |
| History of heart failure | +10 |
| History of chronic lung disease | +10 |
| Pulse ≥110 beats/minute | +20 |
| Systolic blood pressure <100mm Hg | +30 |
| Respiratory rate ≥ 30 breaths/minute | +20 |
| Temperature <36^o^C | +20 |
| Altered mental status† | +60 |
| Arterial oxygen saturation <90%‡ | +20 |

**Pulmonary Embolism Severity Index (PESI) Model**

PESI=pulmonary embolism severity index

*A total point score for each patient is assigned by summing the patient’s age in years and the points for each variable. Risk classes are as follows: Class I ≤65 points; Class II 66-85 points; Class III 86-105 points; Class IV 106-125 points; Class V >125 points. Low risk patients are in Class I/II and high risk is III-V

†Defined as disorientation, lethargy, stupor or coma

‡Defined with and without the administration of supplemental oxygen

| **Variable** | **sPESI Score*** |
| --- | --- |
| Age >80 years | 1 |
| History of cancer | 1 |
| Chronic cardiopulmonary disease | 1 |
| Pulse ≥110 beats/minute | 1 |
| Systolic blood pressure <100mm Hg | 1 |
| Arterial oxygen saturation <90%† | 1 |

**Simplified Pulmonary Embolism Severity Index (sPESI) Model**

sPESI=simplified pulmonary embolism severity index

*A total point score for each patient is assigned by summing the points for each variable. The score corresponds to risk classes: Low risk = 0, High risk ≥1

†Defined with and without the administration of supplemental oxygen

| **Variable** | **Hestia Criteria Score*** |
| --- | --- |
| Hemodynamically unstable | 1 |
| Thrombolysis or embolectomy needed | 1 |
| High risk for bleeding† | 1 |
| Oxygen needed to maintain a PaO2>90% for >24 hours | 1 |
| Pulmonary embolism diagnosed during anticoagulant treatment | 1 |
| Intravenous pain medication for >24 hours | 1 |
| Medical or social reason for treatment in the hospital >24 hours | 1 |
| Creatinine clearance <30mL/minute | 1 |
| Severe liver impairment‡ | 1 |
| Pregnant | 1 |
| History of heparin-induced thrombocytopenia | 1 |

**Hestia Criteria**

PaO2=arterial oxygen saturation

*A total point score for each patient is assigned by summing the points for each variable. If the score >0, the patient cannot be treated at home (score=o signifies low-risk)

†Gastrointestinal bleeding in the preceding 14days, recent stroke (less than 4 weeks ago), recent operation (less than 2 weeks ago), bleeding disorder or thrombocytopenia (platelet count < 75x10^9^/L), uncontrolled hypertension (systolic blood pressure > 180 mm Hg or diastolic >110 mm Hg)

‡Assumed present in this study if total bilirubin ≥2.5 mg/dL

| **Risk Factor**^*^ | **Total Cohort,**  **N (%)**  **N=573** | **IMPACT**  **Low-Risk,**  **N (%)**  **N=165** | **IMPACT**  **Higher-Risk,**  **N (%)**  **N=408** | **PESI**  **Low-Risk,**  **N (%)**  **N=218** | **PESI**  **Higher-Risk,**  **N (%)**  **N=355** | **sPESI**  **Low-Risk,**  **N (%)**  **N=177** | **sPESI**  **Higher-Risk,**  **N (%)**  **N=396** | **Hestia**  **Low-Risk,**  **N (%)**  **N=160** | **Hestia**  **Higher-Risk,**  **N (%)**  **N=413** |
| --- | --- | --- | --- | --- | --- | --- | --- | --- | --- |
| Age (years, mean±SD) | 64.4±16.54 | 46.3±11.35 | 71.8±12.09 | 52.0±14.5 | 72.1±12.63 | 56.2±15.04 | 68.1±15.85 | 59.4±17.31 | 66.4±15.84 |
| >80 years | 104 (18.2) | 0 (0) | 104 (25.5) | 6 (2.8) | 98 (27.6) | 0 (0) | 104 (26.3) | 21 (13.4) | 83 (20.1) |
| Male gender | 260 (45.4) | 81 (49.1) | 179 (43.9) | 93 (42.7) | 167 (47.0) | 93 (52.5) | 167 (45.4) | 69 (43.1) | 191 (46.2) |
| Cancer | 178 (31.1) | 12 (7.3) | 166 (40.7) | 9 (4.1) | 169 (47.6) | 0 (0) | 178 (44.9) | 42 (26.3) | 136 (32.9) |
| Cancer (ICD-9) | 106 (18.5) | 0 (0) | 106 (26.0) | 6 (2.8) | 100 (28.2) | 1 (0.6) | 105 (26.5) | 19 (11.9) | 87 (21.1) |
| Chronic cardiopulmonary disease | 158 (27.6) | 20 (12.1) | 138 (33.8) | 30 (13.8) | 128 (36.1) | 0 (0) | 158 (39.9) | 29 (18.1) | 129 (31.1) |
| Chronic lung disease | 135 (23.6) | 19 (11.5) | 116 (28.4) | 29 (13.3) | 106 (29.9) | 0 (0) | 135 (34.1) | 26 (16.3) | 109 (26.4) |
| Chronic lung disease (ICD-9) | 151 (26.4) | 16 (9.7) | 135 (33.1) | 34 (15.6) | 117 (33.0) | 5 (2.8) | 146 (36.9) | 28 (17.5) | 123 (29.8) |
| Heart failure | 38 (6.6) | 1 (0.6) | 37 (9.1) | 1 (0.5) | 37 (10.4) | 0 (0) | 38 (9.6) | 3 (1.9) | 35 (8.5) |
| Heart failure (ICD-9) | 51 (8.9) | 0 (0) | 51 (12.5) | 6 (2.8) | 45 (12.7) | 4 (2.3) | 47 (11.9) | 6 (3.8) | 45 (10.9) |
| Altered mental status at presentation | 29 (5.1) | 3 (1.8) | 26 (6.4) | 0 (0) | 29 (8.2) | 4 (2.3) | 25 (6.3) | 4 (2.5) | 25 (6.1) |
| Cognitive impairment (ICD-9) | 42 (7.3) | 0 (0) | 42 (10.3) | 5 (2.3) | 37 (10.4) | 4 (2.3) | 38 (9.6) | 7 (4.4) | 35 (8.5) |
| Pulse (beats/min, mean±SD) | 92.7±19.40 | 94.2±18.01 | 92.1±19.91 | 91.8±17.68 | 93.2±20.39 | 85.9±13.28 | 95.6±20.90 | 87.6±18.21 | 94.6±19.51 |
| Pulse ≥ 110 beats/min | 124 (21.6) | 40 (24.2) | 84 (20.6) | 33 (15.1) | 91 (25.6) | 0 (0) | 124 (31.3) | 23 (14.4) | 101 (24.5) |
| Systolic blood pressure (mmHg, mean±SD) | 134.2±23.66 | 133.1±22.13 | 134.6±24.27 | 135.6±21.94 | 133.3±24.64 | 137.5±21.89 | 132.7±24.29 | 139.9±22.64 | 131.9±23.66 |
| Systolic blood pressure <100 mmHg | 27 (4.7) | 7 (4.2) | 20 (4.9) | 3 (1.4) | 24 (6.8) | 0 (0) | 27 (6.8) | 2 (1.3) | 25 (6.1) |
| O2 saturation (%, mean±SD) | 96.3±3.44 | 97.0±3.13 | 96.1±3.53 | 97.0±2.51 | 95.9±3.86 | 97.0±2.50 | 96.0±3.76 | 97.2±2.20 | 96.0±3.77 |
| O2 saturation <90% | 25 (4.4) | 4 (2.4) | 21 (5.1) | 1 (0.5) | 24 (6.8) | 0 (0) | 25 (6.3) | 0 (0) | 25 (6.1) |
| Oxygen needed to maintain O2 saturation >90% for >24 hrs | 303 (59.9) | 63 (38.2) | 240 (58.8) | 87 (39.9) | 216 (60.8) | 68 (34.4) | 235 (59.3) | 0 (0) | 303 (73.4) |
| Respiratory rate (breaths/min, mean±SD) | 19.1±3.40 | 18.6±2.51 | 19.4±3.69 | 18.6±2.31 | 19.5±3.89 | 18.7±2.74 | 19.4±3.65 | 18.2±1.95 | 19.5±3.76 |
| Respiratory rate ≥30 breaths/min | 13 (2.3) | 1 (0.6) | 12 (2.9) | 0 (0) | 13 (3.7) | 2 (1.1) | 11 (2.8) | 1 (0.6) | 12 (2.9) |
| Temperature (degrees Celsius, mean±SD) | 97.6±1.36 | 97.8±1.25 | 97.6±1.40 | 97.9±1.23 | 97.5±1.40 | 97.6±1.31 | 97.6±1.38 | 97.5±1.23 | 97.7±1.40 |
| Temperature <36 degrees Celsius | 145 (25.3) | 33 (20.0) | 112 (27.5) | 29 (13.3) | 116 (32.7) | 46 (26.0) | 99 (25.0) | 41 (25.6) | 104 (25.2) |
| Thrombolysis or embolectomy>48 hrs | 2 (0.3) | 1 (0.6) | 1 (0.2) | 1 (0.5) | 1 (0.3) | 1 (0.6) | 1 (0.3) | 1 (0.6) | 1 (0.2) |
| High risk of bleeding^†^ | 62 (10.8) | 22 (13.3) | 40 (9.8) | 27 (12.4) | 35 (9.9) | 38 (9.6) | 24 (13.6) | 0 (0) | 62 (15.0) |
| PE on anticoagulation | 37 (6.5) | 9 (5.5) | 28 (6.9) | 10 (4.6) | 27 (7.6) | 7 (4.0) | 30 (7.6) | 0 (0) | 37 (9.0) |
| History of heparin-induced thrombocytopenia | 3 (0.5) | 1 (0.6) | 2 (0.5) | 2 (0.6) | 1 (0.5) | 1 (0.6) | 2 (0.5) | 0 (0) | 3 (0.7) |
| Medical or social reason for admission^‡^ | 151 (26.4) | 24 (14.5) | 127 (31.1) | 37 (17.0) | 114 (32.1) | 25 (14.1) | 126 (31.8) | 0 (0) | 151 (36.6) |
| Need for intravenous pain medication for>24 hrs | 45 (7.9) | 27 (16.4) | 18 (4.4) | 31 (14.2) | 14 (3.9) | 17 (9.6) | 28 (7.1) | 0 (0) | 45 (10.9) |
| Severe liver impairment^§^ | 5 (0.9) | 3 (0.7) | 2 (1.2) | 2 (0.9) | 3 (0.8) | 2 (1.1) | 3 (0.8) | 0 (0) | 5 (1.2) |
| Liver disease (ICD-9) | 3 (0.5) | 0 (0) | 3 (0.7) | 1 (0.5) | 2 (0.6) | 1 (0.6) | 2 (0.5) | 0 (0) | 3 (0.7) |
| Creatinine clearance <30mL/min | 20 (3.5) | 0 (0) | 20 (4.9) | 14 (3.9) | 6 (2.8) | 15 (3.8) | 5 (2.8) | 0 (0) | 20 (4.8) |
| Renal failure (ICD-9) | 39 (6.8) | 1 (0.6) | 38 (9.3) | 10 (4.6) | 29 (8.2) | 10 (5.6) | 29 (8.2) | 4 (2.5) | 35 (8.5) |
| Hemodynamically unstable^ǁ^ | 63 (11.0) | 15 (9.1) | 48 (11.8) | 19 (8.7) | 44 (12.4) | 9 (5.1) | 54 (13.6) | 0 (0) | 63 (15.3) |
| Myocardial infarction (ICD-9) | 21 (3.7) | 2 (1.2) | 19 (4.7) | 7 (3.2) | 14 (3.9) | 9 (5.1) | 12 (3.0) | 2 (1.3) | 19 (4.6) |
| Cerebrovascular disease (ICD-9) | 6 (1.0) | 0 (0) | 6 (1.5) | 2 (0.9) | 4 (1.1) | 1 (0.6) | 5 (1.3) | 0 (0) | 6 (1.5) |
| Prior major bleeding (ICD-9) | 23 (4.0) | 1 (0.6) | 22 (5.4) | 7 (3.2) | 16 (4.5) | 4 (2.3) | 19 (4.8) | 2 (1.3) | 21 (5.1) |
| Atrial fibrillation (ICD-9) | 55 (9.6) | 2 (1.2) | 53 (13.0) | 11 (5.0) | 49 (12.4) | 6 (3.4) | 49 (12.4) | 13 (8.1) | 42 (10.2) |
| Coagulopathy (ICD-9) | 25 (4.4) | 2 (1.2) | 23 (5.6) | 7 (3.2) | 18 (5.1) | 8 (4.5) | 17 (4.3) | 3 (1.9) | 22 (5.3) |

**eTable 1. Characteristics of Pulmonary Embolism Patients in the 30-Day Mortality Cohort**hrs= hours; ICD-9=International Classification of Diseases-Ninth Revision; min=minutes; N=number; SD= standard deviation; SpO2=oxygen saturation
*Of the 573 patients, 3 (0.5%) patients had unknown values for respiratory rate; 2 (0.3%) for pulse, systolic blood pressure, SpO2, and temperature; 380 (66.3%) for bilirubin (component of liver disease); 1 (0.2%) for platelets (component of bleed risk); and no patients had missing values for glomerular filtration rate (creatinine clearance estimate).
†Gastrointestinal bleeding in the preceding 14 days, stroke in the preceding 4 weeks, procedure in the preceding 2 weeks, bleeding disorder or thrombocytopenia (platelet count < 75 × 109/L), or uncontrolled hypertension (systolic blood pressure > 180 mmHg or diastolic blood pressure > 110 mmHg)
‡ Medical or social reason for treatment as determined by manual chart review
§ Cirrhosis or bilirubin > 2.5 mg/dL
ǁ Pulse≥100 beats/minute and systolic blood pressure ≤ 100 mmHg or condition requiring admission to an intensive care unit

| **Patient** | **Age, years**  **Gender** | **PESI Risk**  **(score)** | **PESI Risk**  **Explanation** | **sPESI Risk**  **(score)** | **sPESI Risk**  **Explanation** | **Hestia Risk**  **(score)** | **Hestia Risk**  **Explanation** | **IMPACT Risk**  **(% estimated)** | **IMPACT Risk**  **Explanation** |
| --- | --- | --- | --- | --- | --- | --- | --- | --- | --- |
| 1 | 33 M | High  (93) | Male;  Heart failure;  ↑RR;  ↑HR | High  (2) | CCPD;  ↑HR | High  (5) | Severe liver disease;  PE on anticoagulation;  IV pain medication >24h;  O2>24h;  Acute heart failure exacerbation  and pneumonia^*^ | Low  (1.2%) | Heart failure |
| 2 | 54 F | Low  (84) | CLD;  ↑HR | High  (2) | CCPD;  ↑HR | High  (3) | O2>24h;  High bleeding risk;  Pulmonary hypertension^*^ | High  (2.8%) | CLD;  Coagulopathy† |
| 3 | 48 F | Low  (78) | Metastatic cancer | High  (1) | Metastatic cancer | High  (1) | Metastatic  cancer^*^ | High  (2.9%) | Metastatic cancer |

**eTable 2. Description of Patients Whom Died In-Hospital and Had Discordant Risk Categorization**

Patient 1 was categorized as low-risk by IMPACT but high-risk by other scales, this is likely because IMPACT does not utilize clinical data for risk assessment. Patients 2 and 3 were marginally below the 85-point cut-off threshold for being classified as high-risk using PESI. Both patients were relatively young females with comorbidities known to increase pulmonary embolism mortality; demonstrating the influence that gender and age can have on this prediction rule.

CCPD=chronic cardiopulmonary disease; CLD= chronic lung disease; HR=heart rate; IV= intravenous; O2=oxygen; PE=pulmonary embolism; RR=respiratory rate

*Describes the qualifying medical/social reason for admission for Hestia and occurred within the first 24 hours

†ICD-9 code 287.31 (Idiopathic thrombocytopenic purpura)

| **Patient** | **Age, years**  **Gender** | **PESI Risk**  **(score)** | **PESI Risk**  **Explanation** | **sPESI Risk**  **(score)** | **sPESI Risk**  **Explanation** | **Hestia Risk**  **(score)** | **Hestia Risk**  **Explanation** | **IMPACT Risk**  **(% estimated)** | **IMPACT Risk**  **Explanation** |
| --- | --- | --- | --- | --- | --- | --- | --- | --- | --- |
| 1 | 54 F | Low  (84) | CLD;  ↑HR | High  (2) | CCPD;  ↑HR | High  (3) | O2>24h;  High bleeding risk;  Pulmonary hypertension^*^ | High  (2.8%) | CLD;  Coagulopathy† |
| 2 | 48 F | Low  (78) | Metastatic  cancer | High  (1) | Metastatic cancer | High  (1) | Metastatic  cancer^*^ | High  (2.9%) | Metastatic cancer |
| 3 | 75 F | Low  (85) | CLD | High  (1) | CCPD | High  (1) | O2>24h | High  (2.9%) | CLD |
| 4 | 63 M | High  (93) | Male  $\downarrow$Temp | Low  (0) | No qualifying  variables | High  (2) | O2>24h;  Intubation due to respiratory failure* | Low  (1.48%) | No qualifying  variables |

**eTable 3. Description of Patients Whom Died After Discharge and Within 30-days of Presentation and Had Discordant Risk Categorization**

Patients 1, 2, and 3 were marginally below the 85-point cut-off threshold for being classified as high-risk using PESI. The three patients had comorbidities known to increase mortality in pulmonary embolism patients and were classified as high-risk by all other prediction rules. Patient 4 had no relevant comorbidities and was classified as low-risk by IMPACT; however, this patient’s estimated risk was only marginally below the 1.5% threshold for high-risk by IMPACT. Patient 4 was also classified as low-risk by sPESI.

CCPD=chronic cardiopulmonary disease; CLD= chronic lung disease; HR=heart rate; O2=oxygen; Temp= temperature

*Describes the qualifying medical/social reason for admission for Hestia and occurred within the first 24 hours

†ICD-9 code 287.31 (Idiopathic thrombocytopenic purpura)
